# The Potential Roles of Oral Hypoglycemic Agents to Modulate Mitochondrial Function in Type 1 Diabetes Mellitus: A Scoping Review

**DOI:** 10.3390/life16071135

**Published:** 2026-07-08

**Authors:** Su-Ann Cheng, Jeong Hoon Lim

**Affiliations:** 1 Department of Medicine, National University Hospital, 5 Lower Kent Ridge Road, Singapore 119074, Singapore; suann.cheng@mohh.com.sg; 2 Department of Medicine, Yong Loo Lin School of Medicine, National University of Singapore, 1E Kent Ridge Road, Singapore 119228, Singapore

**Keywords:** type 1 diabetes mellitus, oral hypoglycemic agent, mitochondria

## Abstract

Type 1 diabetes mellitus (T1DM) is characterized by autoimmune β-cell destruction and absolute insulin deficiency. While insulin remains the cornerstone of treatment, the adjunctive use of oral hypoglycemic agents (OHAs) has been explored, though clinical evidence in T1DM remains sparse. Mitochondrial dysfunction is increasingly recognized in the pathogenesis and complications of T1DM, and some OHAs are known to modulate mitochondrial pathways, primarily studied in type 2 diabetes mellitus. This review aimed to synthesize existing evidence regarding the roles of OHAs in T1DM, with a specific focus on their potential impact on mitochondrial function. Following PRISMA guidelines, eligible studies investigating mitochondrial dysfunction in T1DM or the effects of OHAs on mitochondrial function in T1DM were included. Of 997 articles screened, 24 studies met inclusion criteria. Twenty studies described the mechanisms of mitochondrial dysfunction in T1DM, highlighting oxidative stress, impaired ATP production, disrupted proteostasis, apoptosis, and altered mitochondrial dynamics. Four preclinical studies suggested that metformin and empagliflozin may improve mitochondrial quality control in an adenosine monophosphate-activated protein kinase (AMPK)-dependent manner by enhancing biogenesis and preventing mitochondrial fission in T1DM. Certain OHAs may modulate mitochondrial dysfunction in T1DM, but clinical translation remains speculative and requires further investigation regarding their potential as adjunctive therapy.

## 1. Introduction

Type 1 diabetes mellitus (T1DM) is caused by the autoimmune destruction of pancreatic β-cells, leading to an absolute deficiency in insulin production [1]. While insulin therapy remains the cornerstone of T1DM management—as emphasized in the 2024 American Diabetes Association guidelines [2]—intensive insulin regimens frequently fail to prevent long-term complications and are inherently limited by risks of severe hypoglycemia and weight gain. Consequently, there remains a compelling clinical need for adjunctive therapies that can stabilize glycemic variability and preserve residual β-cell function.

There is growing interest in the potential benefits of certain oral hypoglycemic agents (OHAs) for this population. Emerging evidence suggests that some OHAs may reduce insulin requirements, improve glycemic variability, delay progression, and potentially mitigate diabetes-related complications in T1DM [3,4]. For example, Dipeptidyl peptidase-4 (DPP-4) inhibitors may enhance endogenous incretin activity, improve β-cell function, and attenuate autoimmunity to potentially delay progression [3,5]. Metformin may be effective in patients with impaired insulin sensitivity by reducing insulin dose, body weight, and low-density lipoprotein cholesterol (LDL-C), although sustained glycated hemoglobin (HbA1c) benefit has not been consistently demonstrated [6]. Glucagon-like peptide-1 (GLP-1) receptor agonists primarily reduce postprandial blood glucose and insulin dose through delayed gastric emptying, appetite suppression, and reduced glucagon secretion, but their use in T1DM is limited by gastrointestinal adverse effects and concerns regarding hypoglycemia or ketosis [7]. Sodium–glucose cotransporter 2 (SGLT2) inhibitors improve glycemic control through insulin-independent urinary glucose excretion and may reduce HbA1c, body weight, blood pressure, and insulin dose; however, their use is constrained by the increased risk of diabetic ketoacidosis, including euglycemic ketoacidosis [8,9].

Thus, while these agents may provide metabolic benefits, their long-term efficacy and safety in T1DM remain uncertain. Among adjuvant therapies, pramlintide, a synthetic analog of the pancreatic hormone amylin, is currently the only non-insulin agent approved for adjunctive use in T1DM [4,10]. However, the clinical use of OHAs, such as metformin and SGLT2 inhibitors, is not routinely recommended due to safety concerns—particularly the increased risk of diabetic ketoacidosis [2]—and limited long-term efficacy data in T1DM. Therefore, clinicians and patients face a dilemma: while adjunctive metabolic protection is needed, current OHA options lack robust data regarding therapeutic efficacy and safety profiles.

Recently, mitochondrial dysfunction has emerged as an important contributor to the pathogenesis and progression of T1DM. Mitochondria play a central role in cellular energy metabolism, apoptosis regulation, immune cell activation, and metabolic homeostasis [11]. Abnormalities in mitochondrial oxidative phosphorylation, increased reactive oxygen species generation, and altered mitochondrial dynamics have been described in pancreatic β-cells, skeletal muscle, and immune cells. Consequently, mitochondrial impairment may exacerbate β-cell loss and immune dysregulation [12], thereby contributing to disease progression and metabolic inflexibility.

Importantly, several OHAs exert pleiotropic effects beyond glucose lowering and have been shown to modulate mitochondrial pathways, including mitochondrial respiration, biogenesis, and oxidative stress signaling, in various metabolic conditions. However, a major gap in current clinical translation exists: while such mitochondrial effects are relatively well characterized in type 2 diabetes mellitus (T2DM), T1DM-specific data remain limited and fragmented. A clear consensus on the extent to which these mechanisms translate into clinically meaningful benefits or risks in T1DM is currently lacking.

To address these unresolved clinical issues, this scoping review aims to comprehensively examine the existing literature on the role of OHAs in T1DM, with a particular focus on their relationship with mitochondrial dysfunction. By integrating molecular insights with available clinical evidence, this review seeks to bridge the gap between mitochondrial biology and bedside application. Through this approach, we aim to update the current understanding of mitochondrial dysfunction in T1DM and explore whether OHAs can be repurposed to offer therapeutic benefits through mitochondrial pathways, thereby identifying knowledge gaps, safety thresholds, and potential clinical implications to guide further research.

## 2. Methods

### 2.1. PRISMA Guidelines

This scoping review was conducted in accordance with the Preferred Reporting Items for Scoping Reviews and Meta-Analyses (PRISMA) guidelines [13]. The completed PRISMA checklist is provided. Given the limited and heterogeneous research specifically addressing the role of OHAs in mitochondrial dysfunction in T1DM, this scoping review focuses on mapping and summarizing the available evidence rather than performing a formal methodological quality assessment or critical appraisal of individual studies.

### 2.2. Literature Search

A comprehensive literature search was performed across multiple electronic databases, including MEDLINE (via PubMed), SCOPUS, BIOSIS and Web of Science, covering all records from inception up to 5 December 2025. The search was limited to articles published in English. The search strategy employed combinations of keywords, including “type 1 diabetes mellitus,” “mitochondria,” and “oral hypoglycemic,” as well as more specific combinations, such as

“T1DM” AND “mitochondria”.

“oral hypoglycemic” AND “T1DM”; “metformin” AND “T1DM”; “SGLT2 inhibitor” AND “T1DM”; “GLP-1 receptor agonist” AND “T1DM”; “Thiazolidinedione” AND “T1DM”; “sulfonylurea” AND “T1DM”; “DPP-4 inhibitor” AND “T1DM”; “Imeglimin” AND “T1DM”.

**Figure 1:**
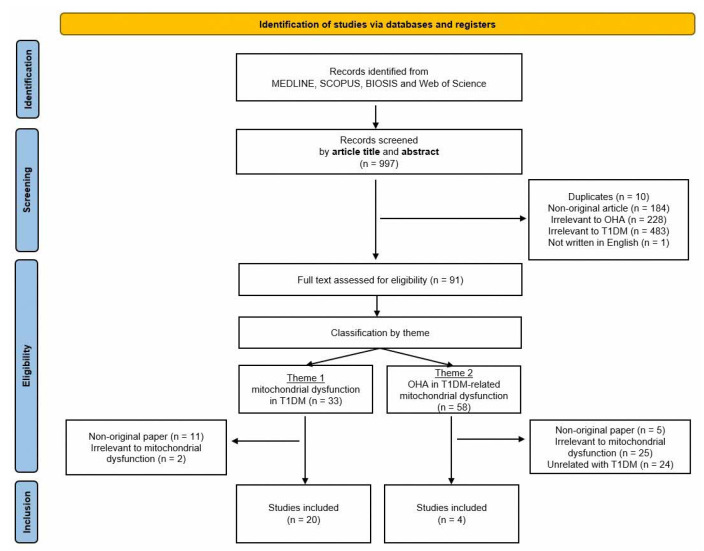
A PRISMA flow diagram of the study selection process. The flowchart delineates the systematic search across MEDLINE, SCOPUS, BIOSIS, and Web of Science. It details the identification, screening, and exclusion criteria applied to arrive at the 24 final studies included in the scoping review, categorized by those addressing T1DM mitochondrial pathology (theme 1) and those evaluating OHA interventions (theme 2).

Two independent reviewers (CSA and LJH) screened the titles and abstracts of all retrieved records to assess their relevance to the study objectives. Records deemed potentially eligible based on keywords, study population, or mitochondrial outcomes were advanced to full-text review. Full-text articles were classified by two themes, mitochondrial dysfunction in T1DM (theme 1) and OHA in T1DM-related mitochondrial dysfunction (theme 2). Any disagreements at each stage were resolved through discussion until consensus was reached. The full search strategy is summarized in Figure 1.

### 2.3. Data Extraction

Data extraction was performed independently by the two reviewers using a standardized data collection form. Extracted information included the year of study, study design, population characteristics, type of OHAs evaluated, mitochondrial pathway studied, and key findings related to mitochondrial function and T1DM.

### 2.4. Inclusion and Exclusion Criteria

Inclusion criteria:

- Original research paper.
- Investigating the relationship between T1DM and mitochondrial function.
- Investigating the roles of OHAs on mitochondrial function in T1DM.

Exclusion criteria:

- Duplicates.
- Non-original research paper: review article, editorial, book chapter, meta-analysis, case report, conference abstract without full data.
- Paper unrelated to mitochondrial function.
- Paper unrelated to OHAs.
- Paper unrelated to T1DM.
- Paper not published in English.

For consistency with the scope of this review, only oral GLP-1 receptor agonist formulations were considered eligible, while injectable GLP-1 receptor agonists were excluded from the formal evidence synthesis. Review articles, editorials, book chapters, meta-analyses, and case reports were excluded to preserve the objectivity of the synthesis, avoid data redundancy, and ensure the map reflects only primary empirical evidence.

## 3. Results

### 3.1. Selection of Studies

An automated literature search utilizing search queries across multiple electronic databases sorted out a total of 997 articles. We assessed eligibility by checking titles and abstracts, excluding 906 articles according to the criteria: 10 duplicates; 184 non-original articles; 228 papers not related to OHAs, such as those focusing on insulin, herbs or traditional medicines; 483 articles irrelevant to T1DM; and 1 article not written in English. The full texts were retrieved for the remaining 91 articles and were classified into two themes: 33 papers regarding mitochondrial dysfunction in T1DM (theme 1) and 58 papers regarding the roles of OHAs in T1DM-related mitochondrial dysfunction (theme 2). Upon reviewing the full-text articles, we removed papers irrelevant to mitochondrial function, such as those investigating mitochondrial morphology or mitochondrial genomics, etc. At this step, 13 papers were excluded from theme 1 and 54 papers from theme 2, as illustrated in Figure 1. Ultimately, 24 relevant papers published between 1986 and 2025 were included. Among them, 21 papers [9,10,11,12,13,14,15,16,17,18,19,20,21,22,23,24,25,26,27,28] addressed mitochondrial dysfunction in T1DM, and 4 papers [29,30,31,32] investigated the role of OHAs in mitochondrial dysfunction in T1DM. To note, we identified T1DM animal studies based on the description of the experimental methodology regarding a streptozotocin-induced diabetic animal model [32]. The searches of clinical trial registries did not identify any additional eligible studies. A summary of the overall selection process is presented in a PRISMA flowchart (Figure 1).

### 3.2. Study Characteristics

Twenty studies [14,15,16,17,18,19,20,21,22,23,24,25,26,27,28,29,30,31,32,33] that addressed mitochondrial dysfunction in T1DM are summarized in Table 1. Fourteen studies [14,15,17,20,21,23,25,26,27,28,29,30,31,33] primarily involved in vivo animal studies. Among these, most used STZ-induced diabetic models, including mice [14,27,32,33], rats [15,17,20,21,26,29,30,31], and one canine model [28], while one study used non-obese diabetic (NOD) mice, which spontaneously develop autoimmune β-cell destruction resembling T1DM [25]. Four studies involved human T1DM samples [16,18,19,24], including peripheral blood mononuclear cell analysis, observational cross-sectional studies, and transcriptomic or in silico analyses. Two studies [22,23] used in vitro insulin-producing cell or isolated islet models to investigate cytokine-induced mitochondrial stress and β-cell injury. One study [32] combined in vivo and in vitro approaches to explore mitochondrial complex I inhibition and β-cell apoptosis.

Due to the paucity of literature specifically addressing the roles of OHAs in T1DM-related mitochondrial dysfunction, only four preclinical studies [34,35,36,37] were identified, as summarized in Table 2. Among these, two classes of OHAs were investigated: biguanides (metformin) in two studies [34,36] and SGLT2 inhibitors (empagliflozin) in the other two studies [35,37].

### 3.3. Mitochondrial Dysfunction in T1DM

The included studies highlighted several key pathological mechanisms of mitochondrial dysfunction in T1DM. Oxidative stress and reactive oxygen species (ROS) overproduction were the most frequently reported pathways [14,16,23,27], with documented elevated mitochondrial ROS and oxidative damage to lipids and proteins.

Impaired bioenergetics and ATP production [16] were reported and characterized by the reduced activity of electron transport chain (ETC) complexes and diminished mitochondrial ATP synthesis. Mitochondria-mediated apoptosis pathways were addressed in seven articles [19,21,24,25,26,32,33], highlighting mitochondrial membrane depolarization, cytochrome c release, and caspase activation. Altered Ca^2+^ homeostasis and an increased susceptibility to mitochondrial permeability transition pore (MPTP) opening have been identified, suggesting that intracellular Ca^2+^ disruptions critically contribute to mitochondrial dysfunctions [15,17,29].

Three articles [21,26,30] reported disrupted mitochondrial proteostasis and protein quality control (PQC), which emphasized the impaired expression of mitochondrial chaperones and proteases, the accumulation of misfolded or oxidized proteins, and PQC system dysfunction. Altered mitochondrial dynamics including mitophagy and fission or fusion imbalances were mentioned in three articles [17,24,31]. Dysfunctional microRNAs (miRNAs) affecting mitochondria where specific miRNAs were shown to regulate mitochondrial metabolism, apoptosis, and oxidative stress were mentioned in one article [19]. A list of relevant studies on mitochondrial dysfunction in T1DM is summarized in Table 1.

### 3.4. Potential Roles of OHGAs in Mitochondrial Function in T1DM

Only four studies [34,35,36,37] investigating the roles of OHAs in T1DM-related mitochondrial dysfunction were identified. All of them were preclinical in nature, incorporating in vivo animal models and in vitro experimental systems. Most studies reported positive effects, including improved mitochondrial quality control, enhanced bioenergetics, and reduced oxidative stress, as summarized in Table 2.

Two experimental studies [35,37] evaluated empagliflozin, which demonstrates protective effects on mitochondrial structure and function in T1DM animal models across different tissues. In human renal proximal tubule cells (hRPTCs) exposed to high-glucose conditions, empagliflozin treatment improves mitochondrial biogenesis and restores the balance of fusion–fission protein expression. By normalizing AMP-to-ATP ratios, restoring AMPK-α phosphorylation, suppressing the phosphorylation of Dynamin-related protein 1 (Drp1), and upregulating Mitofusin 1 (MFN1), empagliflozin appears to play a significant role in maintaining mitochondrial quality control [35]. Similarly, in the myocardial microvasculature, empagliflozin triggers the activation of AMPK by restoring the AMP-to-ATP ratio. The activation of the AMPK pathway by empagliflozin suppresses the phosphorylation of Drp1, which ultimately inhibits mitochondrial fission, leading to reduced oxidative stress, preserved ATP production, and improved mitochondrial integrity and endothelial function [37]. Collectively, these findings suggest that SGLT2 inhibition may exert beneficial mitochondrial effects through the modulation of mitochondrial dynamics and energy homeostasis.

**Figure 2:**
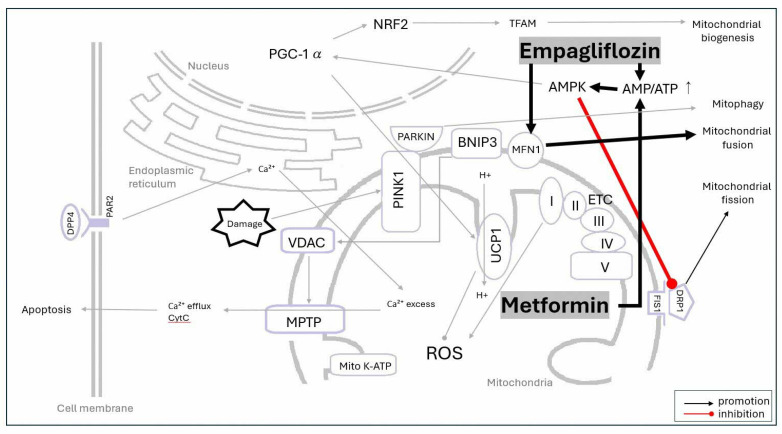
Proposed mechanisms of OHAs in modulating mitochondrial function in T1DM. Schematic representation of how metformin and empagliflozin counteract T1DM-related stress. Key pathways include restoration of AMP/ATP ratio, activation of AMPK, and inhibition of Drp1-mediated mitochondrial fission. These interventions promote mitochondrial biogenesis and restore balance of fusion–fission proteins (e.g., MFN1), ultimately reducing oxidative stress and preserving cellular bioenergetics. Biochemical pathways established for mitochondrial mechanisms in other disease models including T2DM are indicated by thin gray lines.

Metformin, covered in two studies [34,36], has demonstrated mitochondrial protective effects in T1DM animal models. Metformin treatment in hyperglycemic rats has shown beneficial effects on renal energy status, specifically by normalizing the previously depleted levels of ATP and AMP [34]. Metformin reduces mitochondrial ROS production in renal tissue and modulates oxidative stress-related gene expression, conferring protection against diabetic nephropathy [34]. In endothelial cells subjected to high glucose, metformin prevents mitochondrial fragmentation by reducing Drp1 levels and inhibiting its movement into the mitochondria via the AMPK pathway [36]. This inhibition of Drp1-mediated mitochondrial fission leads to reduced mitochondrial fragmentation, decreased oxidative stress, and improved mitochondrial function in endothelial cells. Together, these findings suggest that metformin may ameliorate diabetes-related mitochondrial dysfunction primarily through the attenuation of oxidative stress and modulation of mitochondrial dynamics. However, these findings should be interpreted with caution, as they are derived from a limited body of evidence, primarily from animal models or in vitro studies. The collective mechanistic findings regarding the roles of OHAs in mitochondrial function in T1DM are illustrated in Figure 2.

## 4. Discussion

Mitochondria are essential for normal cell function and generate ATP via oxidative phosphorylation (OXPHOS), regulate ROS levels, modulate apoptosis and maintain calcium homeostasis [38,39]. The disruption of these processes has been increasingly recognized as a key contributor to the pathophysiology of both T1DM and T2DM, although the underlying mechanisms differ. In particular, accumulating evidence suggests that the Ca^2+^-dependent mitochondrial permeability transition pore (MPTP) plays a crucial role in the progression of mitochondrial dysfunction in DM models, which is linked to mitochondrial Ca^2+^ uniporter regulation [15,17,29,40,41]. The formation and opening of the MPTP serve as an early hallmark of mitochondrial stress and perturbation, underscoring its utility as a key biomarker and a potential drug target [42,43]. The excessive accumulation of Ca^2+^ in the mitochondrial matrix triggers the opening of the MPTP in the inner mitochondrial membrane, leading to apoptosis and subsequent cell death, as illustrated in Figure 2.

In pancreatic β-cells, glucose metabolism generates ATP, increasing the ATP/ADP ratio, which closes mitochondrial ATP-dependent potassium (KATP) channels, allows for calcium influx, and triggers insulin secretion. In T2DM, mitochondrial dysfunction is primarily driven by nutrient overload, resulting in elevated ROS production, impaired OXPHOS, and insulin resistance, ultimately contributing to β-cell failure [44]. In contrast, T1DM is characterized by the autoimmune-mediated destruction of β-cells, leading to absolute insulin deficiency [1]. Here, mitochondrial dysfunction arises mainly from cytokine-induced stress, oxidative damage, and apoptosis pathways, rather than nutrient excess. These mechanistic distinctions highlight that findings from T2DM cannot be directly extrapolated to T1DM, emphasizing the need for T1DM-specific investigations.

### 4.1. Potential Roles of OHAs in Modulating Mitochondrial Function in T1DM

Mitochondrial dysfunction in T1DM is characterized by ROS overproduction, impaired ATP synthesis, mitochondria-mediated apoptosis, and disrupted mitochondrial dynamics, all of which contribute to β-cell loss, immune dysregulation, and systemic metabolic impairment, as shown in Table 3. Despite these critical roles, the potential of OHAs to modulate mitochondrial pathways in T1DM remains largely unexplored, representing a significant knowledge gap.

Preclinical studies suggest that agents, such as metformin and empagliflozin in T1DM, may modulate mitochondrial function in renal and endothelial tissues by reducing mitochondrial oxidative stress, improving mitochondrial biogenesis, and restoring the balance of fusion–fission protein expression [34,35,36,37]. It is notable that both metformin and empagliflozin commonly normalize the AMP/ATP ratio, stimulate AMPK, and inhibit Drp1 to improve mitochondrial quality control (MQC) in the renal and endothelial tissues of T1DM models.

In T1DM, insulin therapy remains the cornerstone of treatment, aiming to replenish the insulin absent due to the autoimmune destruction of pancreatic β-cells [1]. Beyond glycemic control, insulin also exerts important effects on mitochondrial function, including the normalization of mitochondrial dynamics and biogenesis, regulation of autophagy, and modulation of tau protein phosphorylation in the brain of T1DM rat models [45]. Insulin also mitigates intracellular Ca^2+^ overload, oxidative stress, and susceptibility to mitochondrial permeability transition pore (MPTP) opening in T1DM rat hearts, particularly when combined with aerobic exercise [17]. Furthermore, alisporivir, a non-immunosuppressive analog of cyclosporin A, was shown to selectively block MPTP opening, thereby exhibiting a hypoglycemic effect and improving mitochondrial structure and function in both in vitro and in vivo models of diabetes [46].

OHAs, widely used in T2DM, similarly modulate mitochondrial function by improving insulin sensitivity and glucose metabolism and in some cases directly restoring mitochondrial bioenergetics [47]. There is growing interest in whether these mitochondrial-modulating properties could have mechanistic relevance in T1DM; however, current evidence is largely preclinical. This is particularly pertinent for individuals with T1DM who develop insulin resistance (“double diabetes”), a condition associated with mitochondrial dysfunction [48,49,50].

Targeting mitochondrial pathways with OHAs may have clinical relevance beyond glycemic control. This is particularly important for T1DM patients with suboptimal glycemia or early microvascular and macrovascular complications, such as nephropathy [51] and neuropathy [52], where insulin alone may not fully address the underlying pathophysiology. MQC imbalance is closely linked to the development of these complications [53]. Given the critical role of MQC in metabolic homeostasis, OHAs could theoretically provide broader pleiotropic benefits, potentially impacting obesity, neurodegenerative conditions, cardiomyopathy, and aging.

### 4.2. Future Research Directions for OHAs in T1DM-Related Mitochondrial Dysfunction

While OHAs are well established in T2DM, their mitochondrial-modulating effects in T1DM remain poorly characterized. Preclinical data suggest that metformin and empagliflozin can reduce oxidative stress and restore mitochondrial dynamics via the AMPK, Drp1, and MFN1 pathways implicated in T1DM. However, it remains largely unexplored how OHAs relate to other mitochondrial processes, such as electron transport chain function, ATP synthesis, mitophagy, and mitochondria-mediated apoptosis (Table 2).

In preclinical or T2DM contexts, imeglimin demonstrated the potential to restore mitochondrial respiratory chain activity, reduce ROS, maintain membrane potential, and suppress hepatic gluconeogenesis through mitochondrial modulation [54,55]. Thiazolidinediones (rosiglitazone, ciglitazone) enhanced the mitochondrial biogenesis and modulated expression of HSP22, oxidative capacity, UCP1 induction, and thermogenic function [56,57]. Sulfonylureas appeared to lower mitochondrial ATP content, increase ADP/AMP levels, and activate the mitochondrial permeability transition pore (MPTP) [58,59,60,61]. High-affinity sulfonylureas (glyburide, glipizide) blocked mitochondrial KATP channels, potentially contributing to adverse cardiovascular events, whereas low-affinity sulfonylurea (glimepiride) preserved mitochondrial integrity under ischemic stress and modulated enzymes affecting gluconeogenesis. Dipeptidyl peptidase-4 (DPP-4) inhibitors induced mitochondrial calcium overload; knockdown studies in cardiomyocytes demonstrated protection against oxidative stress by maintaining bioenergetics, lowering ROS and apoptosis, and enhancing antioxidant signaling [62]. Glucagon-like peptide-1 (GLP-1) receptor agonists restored mitochondrial respiratory chain activity and ATP production, reduced ROS and lipotoxicity, and modulated mitophagy [63,64,65].

Notably, preclinical studies have demonstrated that mitochondria-targeted agents, such as Mito-TEMPO, MitoQ, and SS-31, can confer mitochondrial protection against various conditions, such as acute ischemia–reperfusion injury [66], neurodegenerative disorders [67], acute spinal cord injury [68], traumatic brain injury [69], and T1DM [70,71]. Lipophilic cations intercept free radicals directly, scavenging mitochondrial reactive oxygen species [66,67,68,70,71]. Cardiolipin-binding peptides stabilize mitochondrial crista structure and optimize ETC efficiency, thereby maintaining downstream ion channel activity and halting the metabolic cascade that leads to apoptotic cell death [69]. Given that metformin and empagliflozin reduce oxidative stress, restore mitochondrial dynamics, and improve mitochondrial biogenesis in T1DM models, further investigation into the potential synergistic interactions between OHAs and mitochondria-targeted agents is warranted.

Collectively, these findings highlight the mechanistic potential of OHAs, such as metformin and empagliflozin, to target mitochondrial dysfunction. However, their long-term effects on beta-cells remain unknown, and translating these benefits to T1DM is largely underexplored. Beyond pancreatic beta-cells, critical gaps remain regarding how mitochondrial-modulating OHAs affect other tissues exposed to chronic hyperglycemia. Given that mitochondrial bioenergetics dictate cellular survival and neuroregeneration in secondary complications like diabetic neuropathy, systematic preclinical and translational studies are required. Future research should determine whether these agents restore mitochondrial function in pancreatic beta-cells and other relevant tissues, identify the most promising mitochondrial-targeting OHAs, and evaluate their potential disease-modifying or cell-preserving effects in T1DM. Furthermore, the optimal combination regimens and dose adjustments between OHAs and novel mitochondria-targeted antioxidants remain unmapped, leaving pharmacokinetics to be established. Finally, translating preclinical ex vivo profiles to clinical trials requires robust biomarkers to monitor mitochondrial functional status in vivo. Uncovering these specific bioenergetic metrics will be crucial for patient stratification, ultimately allowing clinicians to identify individuals with T1DM who would benefit the most from targeted metabolic optimization.

### 4.3. Limitations

This scoping review has several limitations. The search strategy may have lacked sensitivity and specificity, potentially omitting relevant studies. Restricting inclusion to published, peer-reviewed articles in English and the lack of systematic gray literature search (e.g., theses, preprints, conference abstracts, and other unpublished work) might have excluded valuable data. This scoping review did not undertake formal critical appraisal or methodological quality assessment, as the aim was to map the breadth of evidence rather than evaluate study rigor.

Most of the research findings presented in this scoping review are derived from mostly preclinical studies and have not been validated in clinical trials. There are only four original papers investigating the role of OHAs in T1DM, which may not be sufficient to translate directly to clinical practice. Thus, any potential protective or nonglycemic benefits of OHAs in T1DM should be interpreted cautiously. OHAs are not routinely used in T1DM, limiting the ability to draw definitive conclusions about mitochondrial effects in T1DM. While these mechanistic insights are informative, OHAs are not currently recommended as standard therapy in T1DM due to the risks of hypoglycemia and ketoacidosis [2].

Despite these limitations, this review highlights mechanistic pathways through which OHAs may influence mitochondrial function in T1DM. Still, it is necessary to conduct extended comprehensive preclinical studies utilizing diverse models and different classes of OHAs to evaluate clinical relevance and impact. Such an approach may help identify the most promising mitochondrial-targeting agents, determine target patient groups, and guide future research to optimize safety and therapeutic benefits.

## 5. Conclusions

Although published data examining OHAs and mitochondrial dysfunction in T1DM remain limited, the studies included in this review—primarily preclinical—highlight the potential roles of metformin and empagliflozin in modulating mitochondrial function. These agents act by reducing mitochondrial oxidative stress, improving mitochondrial biogenesis, and restoring the balance of fusion–fission protein expression in renal and endothelial tissues. While precise mitochondrial pathways require further validation in a tissue- and context-specific manner, understanding the modulating effects of OHA provides insights into potential synergism with other mitochondria-targeted agents via scavenging ROS, maintaining structural components, preserving bioenergetics, and inhibiting apoptosis.

In addition, these findings justify further investigation into other tissues and warrant well-designed preclinical studies to elucidate the mechanistic relationship between diverse OHAs and T1DM-related mitochondrial dysfunction, ultimately guiding the development of adjunctive therapies.

## Figures and Tables

**Table 1:** Summary of studies characterizing mitochondrial dysfunction in T1DM.

Author	Year	Study Design	Study Setting	Control	Study Findings
Baseler WA et al. [14]	2011	In vivo animal study	Laboratory-based research, STZ-induced type 1 diabetic mice to simulate chronic hyperglycemia	Non-diabetic mice receiving vehicle buffer	T1DM causes proteomic alterations primarily in IFM, linked to defective nuclear-encoded protein import, increased oxidative stress from excessive ROS production and contributing to diabetic cardiomyopathy.
Belosludtsev KN et al. [15]	2019	In vivo animal study	Laboratory-based research, STZ-induced type 1 diabetic mice to simulate chronic hyperglycemia	Non-diabetic rats	Diabetic rat liver mitochondria showed ~1.4× higher Ca^2+^ uptake, reduced MCUb, and increased resistance to CsA-sensitive MPT but increased sensitivity to palmitate/Ca^2+^ pores, with altered membrane lipids and higher peroxidation.
Chen J [16]	2017	Human study (3 phases)	PBMC 238 T1DM patients Flowcytometry immunophenotyping and functional study	29 healthy volunteers	T1DM T-cells display intrinsic mitochondrial dysfunction (inner membrane hyperpolarization, altered ATP/ROS/IFN-γ balance) that may drive immune dysregulation and autoimmunity.
Da Silva MF et al. [17]	2015	In vivo animal study	Laboratory-based research, STZ-induced T1DM rats to simulate chronic hyperglycemia	Non-diabetic controls and untreated diabetic models	Both exercise and insulin improve mitochondrial and Ca^2+^ dysfunction in diabetic hearts, but combination therapy is the most effective in reversing oxidative stress and mitochondrial damage.
Dieter C et al. [18]	2019	Human patients with type 1 diabetes	Observational, cross-sectional study with samples analyzed in a molecular biology research laboratory	Healthy individuals without diabetes	miR-30e-5p emerges as a potential biomarker candidate for diabetic kidney disease in T1DM, with measured expression differences and relevant biological connections.
Ferraz RS et al. [19]	2022	In vivo and in silico transcriptomic animal study	Human observational study with transcriptomic and in silico analysis	Healthy controls	Global miRNA expression analysis revealed novel nuclear–mitochondrial interactions in T1DM, suggesting the dysregulation of mitochondrial pathways and potential impact on β-cell function and immune regulation.
Ferreira M et al. [20]	2003	In vivo animal study	Laboratory-based research, STZ-induced type 1 diabetic rats and Goto–Kakizaki rats	Non-diabetic rats	Diabetes induces compensatory metabolic adaptations in rat liver mitochondria, including increased coenzyme Q and cardiolipin levels and reduced susceptibility to mitochondrial permeability transition.
Ferreira R et al. [21]	2013	In vivo animal study	Laboratory-based research, STZ-induced type 1 diabetic rats	Non-diabetic rats	STZ-induced diabetes was associated with cardiac mitochondrial lipidomic alterations, including changes in phospholipid composition that may contribute to mitochondrial dysfunction in diabetic cardiomyopathy.
Gurgul-Convey E et al. [22]	2010	In vitro cell study	RINm5F cells overexpressing PGIS Molecular/cellular physiology laboratory with detailed cellular analyses	Insulin-producing RINm5F cells without PGIS overexpression	Overexpressing PGIS protects insulin-producing β-cells from cytokine-mediated damage by mitigating ER and mitochondrial oxidative stress, highlighting a potential therapeutic strategy to preserve β-cell function in type 1 diabetes.
Gurgul-Convey E et al. [23]	2011	In vitro cell study	Insulin-producing RINm5F cells and isolated pancreatic islets exposed to pro-inflammatory cytokines (IL-1β, TNF-α, IFN-γ) to mimic autoimmune T1DM conditions	IL-1β exposed control cells	Cytokine toxicity in insulin-producing cells is driven by mitochondrial nitro-oxidative stress, with hydroxyl radicals as key effectors; targeting mitochondrial ROS can protect β-cells from inflammatory damage.
Iannantuoni F et al. [24]	2020	Human patients with type 1 diabetes	Observational, cross-sectional study Blood samples obtained from outpatient clinics and processed in a molecular/cellular laboratory	Healthy individuals without diabetes	T1DM is associated with mitochondrial alterations, increased oxidative stress, and enhanced leukocyte–endothelium interactions, which may contribute to cardiovascular complications.
Jelenik T et al. [25]	2014	In vivo animal study	NOD mice, a model that spontaneously develops type 1 diabetes through the autoimmune destruction of pancreatic β-cells	Age-matched non-diabetic mice	In NOD mice, insulin resistance develops in a tissue-specific manner, with early hepatic resistance marked by increased mitochondrial respiration, lipid peroxidation, and JNK activation, as well as stress followed by muscle resistance linked to DAG accumulation and PKCθ activation. Elevated adipose lipolysis and serum fetuin A further exacerbate muscle insulin resistance.
Larsen S et al. [26]	2015	In vivo animal study	Laboratory-based research, STZ-induced hyperglycemia; skeletal muscle (soleus and plantaris) mitochondrial analysis	Control rats given sham citrate buffer injection	STZ-induced chronic hyperglycemia increases intrinsic mitochondrial respiratory capacity in the soleus and plantaris muscles—particularly for lipid- and complex I-linked substrates—despite reduced mitochondrial content, suggesting a compensatory upregulation of mitochondrial function in T1DM models.
Liu HY et al. [27]	2009	In vivo animal study	Laboratory-based research, STZ-induced type 1 diabetic mice to simulate chronic hyperglycemia	Non-diabetic mice	Excess insulin, rather than hyperglycemia, is the dominant driver of insulin resistance in T1DM, suggesting that therapeutic strategies should consider avoiding chronic hyperinsulinemia to prevent tissue-specific insulin resistance. Mitochondrial dysfunction was accompanied by increased ROS generation and oxidative stress, contributing to tissue-specific insulin resistance (particularly in the liver and muscle).
Ma F et al. [28]	2023	In vivo animal study	Veterinary research facility, STZ-induced type 1 diabetic canine to simulate chronic hyperglycemia	Healthy control canine without diabetes	NAC, in conjunction with insulin, offers protective effects in diabetic nephropathy by regulating mitochondrial dynamics and FUNDC1-mediated mitophagy, highlighting its potential therapeutic role in managing diabetic kidney complications.
Oliveria PJ et al. [29]	2003	In vivo animal study	Heart mitochondria isolated from STZ-induced type 1 diabetic rats	Non-diabetic rats	Diabetes increases susceptibility to mitochondrial permeability transition in cardiac mitochondria, causing reduced calcium uptake and depressed oxygen consumption, not due to damage to the calcium uptake machinery but enhanced permeability transition.
Padrão AI et al. [30]	2012	In vivo animal study	Laboratory-based research, STZ-induced type 1 diabetic rats to simulate chronic hyperglycemia	Age-matched, non-diabetic Wistar rats	Compromised mitochondrial protein quality control, characterized by decreased proteolytic activity and increased protein oxidation, contributes to mitochondrial dysfunction in the skeletal muscle of type 1 diabetic rats, which highlights the importance of maintaining mitochondrial proteostasis in preventing diabetic muscle complications.
Silva-Rodrigues T et al. [31]	2020	In vivo animal study	Laboratory-based research, STZ-induced type 1 diabetic rats to simulate chronic hyperglycemia	Age-matched, non-diabetic Wistar rats injected with vehicle (citrate buffer)	Hyperglycemia in a T1DM model induces a reorganization of mitochondrial glucose metabolism and redox balance in the rat brain, which may serve as an early adaptive mechanism to counteract oxidative stress and prevent neurodegeneration associated with mitochondrial complex I deficits.
Wu M et al. [32]	2019	In vivo and in vitro study	STZ-induced T1DM in mice	Healthy male C57BL/6 mice	The inhibition of mitochondrial complex I with rotenone offers protective effects against β-cell apoptosis and oxidative stress and may attenuate T1DM progression, suggesting a potential therapeutic strategy targeting mitochondrial dysfunction.
Zeng Z et al. [33]	2020	In vivo animal study	Laboratory-based research, STZ-induced type 1 diabetic mice to simulate chronic hyperglycemia	Non-diabetic controls and untreated diabetic models	Type 1 diabetes exacerbates intestinal ischemia–reperfusion injury by enhancing inflammation and oxidative stress and activating mitochondrial autophagy. Targeting mitochondrial autophagy pathways may offer potential therapeutic strategies for mitigating intestinal damage in diabetic conditions.

**Table 2:** A summary of studies investigating the role of OHAs in T1DM-related mitochondrial dysfunction.

Authors	Year	OHA	OHA Class	Study Design	Mitochondrial Target	Mitochondrial Function
Alhaider AA et al. [34]	2011	Metformin	Biguanide	In vivo (STZ-induced diabetic rats and normoglycemic rats)	Mitochondrial ROS; AMP/ATP	Restores diabetic nephropathy-induced oxidative stress mRNA levels; normalizes depleted levels of ATP and AMP.
Lee YH et al. [35]	2019	Empagliflozin	SGLT2 inhibitor	In vitro (human renal cell) and in vivo (diabetic mice, renal tissue)	Mitochondrial dynamics (fragmentation, fusion)	Attenuates diabetic tubulopathy by improving mitochondrial biogenesis; upregulates Mitofusin 1 (MFN1). Restores the balance of fusion–fission protein expression.
Wang Q et al. [36]	2017	Metformin	Biguanide	In vivo (STZ-induced diabetic ApoE−/− mice) and in vitro (high glucose-exposed endothelial cells)	Dynamin-related protein 1 (Drp1)	Facilitates AMPK-mediated blockage of Drp1-mediated mitochondrial fission.
Zhou H et al. [37]	2018	Empagliflozin	SGLT2 inhibitor	In vivo (diabetic mice, myocardial tissue)	AMPK activation; Drp1-mediated mitochondrial fission	Reduces mitochondrial fragmentation and ROS. Preserves ATP production; improves mitochondrial integrity and endothelial function.

**Table 3:** Mechanisms of mitochondrial dysfunction in T1DM and corresponding effects of OHAs.

Mitochondrial Dysfunction in T1DM	Suggested OHAs Involved in the Mechanism
Oxidative stress and ROS overproduction [14,17,22,23,24,25,28,30,31,32]	Metformin [34] Empagliflozin [35,37]
Reduced activity of ETC complexes and diminished mitochondrial ATP synthesis [16,18,23,25,26,32,33]	NI
Mitochondria-mediated apoptosis [19,21,24,25,26,32,33]	NI
Altered mitochondrial dynamics [17,24,31]	Empagliflozin [35,37] Metformin [36]
Disrupted proteostasis and PQC [21,26,30]	NI
Dysfunctional miRNAs [19]	NI

NI: No information.

## Data Availability

These data were derived from the following resources available in the public domain: [PUBMED] [https://pubmed-ncbi-nlm-nih-gov.libproxy1.nus.edu.sg/?otool=isgnuslib], [SCOPUS] [https://www-scopus-com.libproxy1.nus.edu.sg/pages/home#basic], [WOS] [https://www-webofscience-com.libproxy1.nus.edu.sg/wos/woscc/basic-search], and [BIOSIS] [https://www-webofscience-com.libproxy1.nus.edu.sg/wos/biosis/basic-search], all accessed on 6 July 2026.

## Abbreviations

The following abbreviations are used in this manuscript:

ADP	Adenosine diphosphate
AMP	Adenosine monophosphate
AMPK	AMP-activated protein kinase
ATP	Adenosine triphosphate
DPP-4	Dipeptidyl peptidase-4
Drp1	Dynamin-related protein 1
ETC	Electron transport chain
FIS1	Fission 1
FUNDC1	FUN14 domain containing 1
GLP-1	Glucagon-like peptide-1
HbA1c	Glycated hemoglobin
IFM	Interfibrillar mitochondria
IFN-γ	Interferon γ
IL-1β	Interleukin 1 β
KATP	ATP-sensitive potassium channel
MCUb	Mitochondrial calcium uniporter b subunit
MFN1	Mitofusin 1
miRNA	microRNA
MPTP	Mitochondrial permeability transition pore
MQC	Mitochondrial quality control
NAC	N-acetylcysteine
NOD	Non-obese diabetic
NRF2	Nuclear respiratory factor 2
OHAs	Oral hypoglycemic agents
PARKIN	E3 ubiquitin ligase
PGC-1α	PPAR γ coactivator 1 α
PGIS	Prostacyclin synthase
PINK1	PTEN-induced kinase 1
PKCθ	Protein kinase C θ
PRISMA	Preferred Reporting Items for Scoping Reviews and Meta-Analyses
PQC	Protein quality control
ROS	Reactive oxygen species
SGLT2	Sodium–glucose cotransporter 2
STZ	Streptozotocin
T1DM	Type 1 diabetes mellitus
T2DM	Type 2 diabetes mellitus
TFAM	Mitochondrial transcription factor A
TNF-α	Tumor necrosis factor α
UCP1	Uncoupling protein 1
VDAC	Voltage-dependent anion channel
